# Black Phosphorus Quantum Dots with Tunable Memory Properties and Multilevel Resistive Switching Characteristics

**DOI:** 10.1002/advs.201600435

**Published:** 2017-03-16

**Authors:** Su‐Ting Han, Liang Hu, Xiandi Wang, Ye Zhou, Yu‐Jia Zeng, Shuangchen Ruan, Caofeng Pan, Zhengchun Peng

**Affiliations:** ^1^ College of Optoelectronic Engineering Shenzhen University Shenzhen 518060 P. R. China; ^2^ Shenzhen Key Laboratory of Laser Engineering College of Optoelectronic Engineering Shenzhen University Shenzhen 518060 P. R. China; ^3^ Beijing Institute of Nanoenergy and Nanosystems Chinese Academy of Sciences Beijing 100083 P. R. China; ^4^ Institute for Advanced Study Shenzhen University Shenzhen 518060 P. R. China

**Keywords:** black phosphorus quantum dots, flexible memory, multilevel datastorage, resistive switching, tunable memory characteristics

## Abstract

**Solution‐processed black phosphorus quantum‐dot‐based resistive random access memory** is demonstrated with tunable characteristics, multilevel data storage, and ultrahigh ON/OFF ratio. Effects of the black phosphorous quantum dots layer thickness and the compliance current setting on resistive switching behavior are systematically studied. Our devices can yield a series of SET voltages and current levels, hence having the potential for practical applications in the flexible electronics industry.

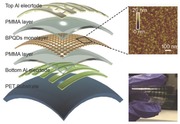

Resistive random access memories (RRAMs), with a typical metal–insulator–metal architecture, exhibit great potential for the construction of future nonvolatile memories because of their high scalability, substantial endurance, long retention time, excellent memory performance, and low operating energy.^1^, ^2^, ^3^, ^4^, ^5^, ^6^ The operation of RRAMs relies on the change in resistance between the high resistance state (HRS) and the low resistance state (LRS). Since the RRAM device can be electrically switched between the HRS and the LRS, corresponding to the “0” and “1” states, respectively, data can be written to and read from the memory device. The ability to tune the memory characteristics, including the control of switching behavior and SET voltage, as well as the access to the multilevel data storage, is imperative for state‐of‐the‐art RRAMs.^7^ The introduction of such capabilities has ushered in a new era of digital logic, analog circuits, nonvolatile memory storage, and neuromorphic computing. Modulating the SET voltage to a reasonable value can both prevent the device from accidently being set by the thermal fluctuation and protect the unselected cells in the display from being programmed.^5^ Precise control of broad switching window permits accurate reading of RRAMs.^8^ Moreover, multibit or multilevel coding paradigms enable more information storage in one unit memory cell and the information can be maintained without consuming power.^9^, ^10^

Current state‐of‐the‐art RRAMs are usually based on insulating oxides, such as TiO_2_, which are limited by the poor external control of the switching voltage as well as the filament formation.^11^, ^12^, ^13^, ^14^ 2D materials, such as transition‐metal dichalcogenides (TMDs) and graphene, are emerging candidates for constructing nonvolatile memory devices. With low power consumption, 2D material‐based memories are good for portable and wearable electronics.^15^, ^16^, ^17^, ^18^ Various studies have investigated 2D nanosheets as a replacement for materials used in conventional floating gates,^19^, ^20^ the semiconductor channel^21^ in the flash memory, and the active layer in RRAM.^8^ Recently, a conceptually novel layered semiconducting material, black phosphorous (BP), with its unique structure and intriguing electronic and optical properties, has become a new focus of 2D material research. Since bulk BP is composed of the puckered layers held together via weak van der Waals forces, BP nanosheets can be conveniently laminated in various ways.^22^, ^23^, ^24^ Compared to indirect‐gap TMDs and zero gap graphene, BP layers are theoretically anticipated to show tunable direct gap from 0.3 to 2 eV with decreasing thickness to a monolayer.^23^ To date, very limited work has been done on BP‐based RRAM devices.^25^ In fact, neither the demonstration of tunable switching behavior and SET voltage nor the realization of multilevel data storage with a high level of control and high sensitivity to compliance current, in BP‐based RRAM has been reported. To address the above critical issues, this study introduces self‐assembled BP quantum dots (BPQDs) sandwiched between two poly(methyl methacrylate) (PMMA) polymer layers as an active layer, and demonstrates that the BPQD–polymer hybrid structure can be engineered to construct RRAMs with distinctively tunable electrical performance. Owing to the quantum confinement and the edge effects, quantum dots (QDs) of 2D materials exhibit novel optical as well as electronic properties in addition to their existing 2D properties.^26^, ^27^ For instance, MoS_2_ QDs and graphene QDs have been successfully prepared and widely used in optoelectronics,^28^ photovoltaic devices,^29^ and biological analysis.^30^

This work is the first to systematically study the ex situ and in situ control of the memory characteristics of BPQD‐based RRAM. In particular, we explore the memory characteristics with respect to device architecture and compliance current values. Our reversible RRAM device exhibits excellent memory performances with an extremely large ON/OFF ratio of 3.0 × 10^7^, which is a record ratio among all the reported 2D material‐based RRAMs to date. By varying the BPQDs trap density, we are able to realize precise ex situ control of the SET voltage and the conductance states of the BPQD‐based resistive switching memory device. Furthermore, we demonstrate robust and reliable in situ control of data storage levels through the modulation of the compliance current. Advantages over other 2D material‐based RRAMs, the plausible mechanisms of the resistive switching behavior, and tunable memory device operation are further discussed.

The 3D illustration and fabrication process of BPQD‐based RRAM are shown in **Figure** **1**a,b. The device began with 100 nm aluminum (Al) patterned on the poly(ethylene terephthalate) (PET) flexible substrates as the parallel bottom electrodes. Then, a PMMA layer was deposited onto the substrate with the spin‐coating technique. Suspension of the BPQDs was initially dispensed onto the PMMA layer for 2 min self‐assembly process, followed by blowing away the suspension with a rubber suction bulb in one direction. After building the self‐assembled BPQD layer, another PMMA layer was spin coated to form resistive switching materials with the sandwich structure. Finally, crossbar array‐based architecture is fabricated by depositing 1 mm × 1 mm top Al electrodes onto the active layer. The atomic force microscopy (AFM) image in Figure 1c, which illustrates the result of drop casting at an optimal concentration of BPQDs, confirms the high quality of the BPQD layer that formed during the self‐assembly process. Shown in Figure 1d is an optical image of the flexible BPQD‐based RRAMs array.

**Figure 1:**
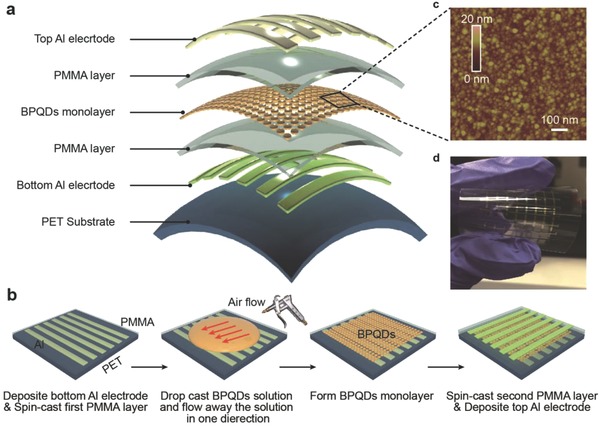
Structure design of BPQD‐based RRAM devices. a) 3D illustration of device structure. b) Schematic diagram depicting the basic fabrication process of flexible BPQD‐based RRAMs. c) Tapping mode AFM height image of the fabricated BPQDs. d) Optical image of the fabricated memory device.

Using a controllable and facile solution‐processed method, we prepared BPQDs from a BP crystal. The solution can stabilize without any obvious aggregation at room temperature for more than 30 d. The low magnification transmission electron microscope (TEM) image in **Figure** **2**a depicts the morphology of BPQDs with the average size of 2.7 ± 0.1 nm (Figure 2e). The (040) and (111) planes of the BPQDs associated with lattice fringes of 0.261 and 0.254 nm were observed in a high‐resolution transmission electron microscope (HRTEM) image, shown in Figure 2b. An enlarged HRTEM image of BPQDs and its corresponding fast Fourier transform (FFT) pattern are displayed in Figure 2c,d, respectively. The BPQDs were further characterized by Raman spectroscope, shown in Figure 2f. The observed characteristic out‐of‐plane phonon mode of A_1g_ at 361.6 cm^−1^ and two in‐plane modes of B_2g_ at 438.7 cm^−1^ and A^2^_g_ at 466.1 cm^−1^ are consistent with those reported in a previous study.^25^ Figure 2g displays optical photographs of the exfoliated BPQDs dispersed in *N*‐methyl‐2‐pyrrolidone (NMP), which present a faint claybank phase.

**Figure 2:**
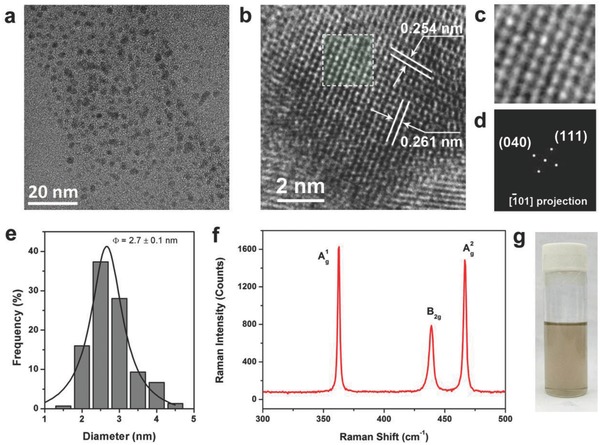
Characterization of BPQDs. a) Low magnification TEM image of BPQDs. b) HRTEM image of a BPQD. c) Enlarged white highlighted part in the HRTEM image. d) Fast Fourier transform (FFT) pattern of a BPQD from (c). e) Statistical analysis of the sizes of 150 BPQDs measured from TEM images. f) Raman characterization of BPQDs. g) Optical image of the BPQDs solution.

On completion of device fabrication, we first investigated the electrical characteristics and switching effects of the BPQD‐based RRAM with PET/Al/35 nm PMMA/3–4 nm BPQDs/35 nm PMMA/Al structure. The typical current–voltage (*I*–*V*) curve is shown in **Figure** **3**a. With the applied voltage sweeping from 0 → +3 → 0 → −3 → 0 V, the measured current value is plotted on a logarithmic scale. A clear bipolar switching with bistable resistance states was observed in memory device. When the device is swept from 0 to 3 V in the positive direction, the current increases gradually with the raising voltage, but then at 2.9 V a sudden increase in current from 6.76 × 10^−9^ to 0.0043 A occurs (stage II), indicating that device switches from the HRS to the LRS. When *V* ≤ 2.9 V, *I* ∝*V^m^* can be utilized to describe Stage I, where *m* exhibits a monotonic increase with respect to the bias (Figure S1, Supporting Information).^17^ This type of dependence implies that the space‐charge‐limited current dominates the carrier transport process, which was also observed in complex‐oxide RRAMs.^31^ The memory device stays in the LRS as the voltage sweeps back to zero, suggesting a nonvolatile memory behavior. The *I–V* curve can be fitted by the Ohmic current model (Figure S2, Supporting Information), with a slope of ≈0.99 following the equation *I* ∝ *V* exp (−Δ*E*_ae_/*kT*), where Δ*E*_ae_ represents electron activation energy. This fitting suggests that the electric field formed in our device can provide sufficient energy for electron transport. During the negative bias sweep, the HRS can be recovered when the reverse voltage reaches −2.6 V. This shift from the LRS to the HRS corresponds to the erasing operation in conventional memory devices. Figure 3b shows the log–log plot for *I–V* characteristics of BPQD‐based RRAM, with clear indication of the different conduction mechanisms in different regions.

**Figure 3:**
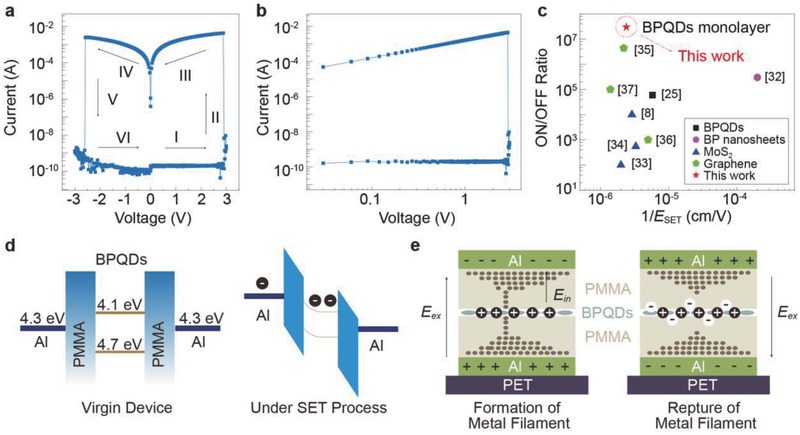
Electrical characterization of BPQD‐based RRAM and switching mechanism. a) Typical current–voltage (*I–V*) plot of PET/Al/35 nm PMMA/BPQDs/35 nm PMMA/Al cells in the “write–read–erase–read” cycle. b) Log–log plot for *I–V* characteristics. c) Comparison of various 2D material‐based RRAMs. d) Schematic band diagram of the virgin device and the device under the writing operation. e) Schematic illustration of resistive switching for device B including formation of filament and rupture of filament.

We obtained a maximum ON/OFF ratio of 3.0 × 10^7^ between the LRS and HRS for BPQD‐based memory device at 2.8 V. To compare the extraordinary resistive switching properties of BPQD‐based RRAMs with those of 2D material‐based resistive switching devices, we plot the ON/OFF ratio versus the reciprocal of the electric field of SET operation (1/*E*_SET_) in Figure 3c. Among BPQD‐PVP‐based RRAM,^25^ pristine BP nanosheets‐based RRAM^32^ and other traditional 2D material composite‐based RRAMs including MoS_2_‐based RRAMs^8^, ^33^, ^34^ as well as graphene and its derivatives based RRAMs,^35^, ^36^, ^37^ our BPQD‐based RRAM yields a record high ON/OFF ratio and a moderate 1/*E*_SET_. This feature guarantees a low misinterpretation rate during the operation of our memory device. The excellent performance may be related to the electronic states in BPQDs.^38^, ^39^

Here, we propose the mechanism of tunable resistive switching behaviors in BPQD‐based RRAM. Since the Ohmic current of the LRS‐induced carrier transport is not subject to current direction, traps‐induced space charge, as well as interface properties, the current in the LRS might drift through the metallic filament.^35^, ^40^ Thus, the resistive switching effect is mainly attributed to the formation of the metallic filament in the hybrid active layer. The alignment of energy band across the device architecture is shown in Figure 3d. A Schottky barrier contact is formed between the interface of the wide bandgap PMMA and Al contact. The Fermi level of Al (work function is 4.3 eV) is very close to the conduction band of the BPQDs (electron affinity is 4.1 eV), which induces an electron injection barrier that is much lower than the hole injection barrier.^41^ Therefore, the electrons injected from the electrodes can be captured by BPQDs trapping sites, while the barrier height and width of dielectric layer are modulated by the applied voltage. Electrons are stored in the valance band of the BPQDs domains surrounded by the bandgap of the PMMA dielectrics, similar to that in the metal nanoparticles floating gate flash memory.^42^ In addition, the strong internal electric field built from the trapped charge carriers may facilitate the formation of metallic filament, which enhances the conduction in the hybrid active layer.

As the charge carriers trapped by the impurity traps in PMMA are not able to build an internal electrical field strong enough to form metallic filaments, it is reasonable that no memory effect was observed in the pristine PMMA‐based RRAM (Figure S3, Supporting Information).^35^ For BPQD‐based memory device, the applied external electrical fields estimated from the equation *E* = *V*/(*d*_1_ + *d*_2_) is 0.48 MV cm^−1^, where *d*_1_ and *d*_2_ are the thicknesses of the first and second PMMA layer, respectively. Such strong electric field increases the injecting efficiency of the electrons and the holes into the charge trapping layer in the SET and RESET process, respectively. The device can be erased back to the HRS because it is easy to release the trapped electrons and rupture the formed metallic phase. The formation and rupture of the filament during the SET and RESET processes of BPQD‐based RRAM are illustrated in Figure 3e. This analysis is in agreement with the observed reversible bipolar resistive switching performance of BPQD‐based RRAM.

The switching cycles of BPQDs‐based RRAM measured with a series of voltage pulses are displayed in **Figure** **4**a and the reliability test of BPQD‐based RRAM with applications of writing and erasing cycles is shown in Figure 4b. SET process with a 2.9 V pulse and RESET process with a −2.6 V pulse, both with a 0.1 s time interval, are used to switch between the HRS and the LRS. The current level of the HRS and the LRS is read by a substhreshold voltage of 0.5 V, which is carefully selected to read the status of various memory levels. This subthreshold voltage pulse is large enough to harvest a measurable current, but unable to adjust the conductance. During 100 cycles under the ambient conditions, the device did not exhibit obvious fluctuation. The retention capability is also measured with respect to eclipsed time, shown in Figure 4c. The ON/OFF ratio does not exhibit significant degradation after 10^4^ s, indicating its excellent stability. Furthermore, bending test was carried out by repetitively bending the devices to a 15 mm radius. The bended device exhibits a bipolar resistance switching behavior similar to that of the flat device. As shown in Figure 4g, almost no bipolar resistance switching degradation occurred after 500 bending cycles, suggesting that the BPQD‐based RRAM is a promising candidate for next‐generation flexible nonvolatile memory device.

**Figure 4:**
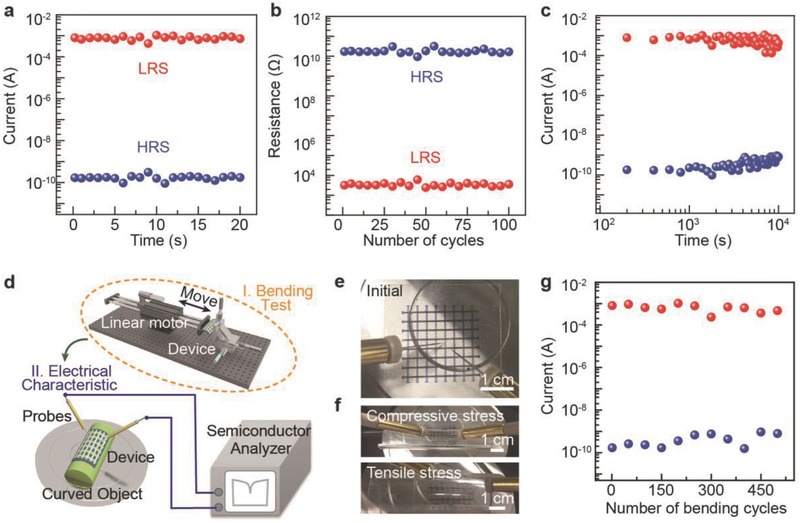
Endurance, retention, and mechanical properties of BPQD‐based memory device. a) Switching cycles of BPQD‐based RRAM measured with voltage pulses. b) Switching endurance of flexible memory device. c) Retention test of BPQD‐based RRAMs in ambient conditions. d) Illustration of experimental setup. Optical image of the device tested in e) flat condition, and f) compressive strain and tensile strain. g) Mechanical stability test of the BPQD‐based RRAMs that was carried out by repeatedly bending the memory devices with a bending radius of 15 mm.

For a practical memory device, the distinct controllability of the SET voltage and the HRS/LRS ratio based on the switchable and sustainable resistance is the most important requirement. The SET voltage and the current level within a device can be modulated by varying the ex situ thickness of the BPQD layer and the in situ compliance values. Tapping mode AFM images of 3–4, 12, and 20 nm BPQD layers with height profiles are displayed in **Figure** **5**a–c. The memory performance of various thicknesses of the BPQD layer is shown in Figure 5d. Sweeps of 0 → +3 → 0 → −3 → 0 V are applied on the memory devices. Clearly, the SET voltage of the devices is reduced with increasing thickness of the BPQD layer. By increasing the thickness of BPQD from 3–4 to 20 nm, the turn‐on voltage decreases from 2.9 to 1.9 V in the positive sweep and the turn‐off voltage decreases from −2.6 to −1.7 V in the negative scan. The HRS of a memory device with 3–4 nm thick of the BPQD layer is in the insulating state, with a resistance of 10^9^–10^10^ Ω, while the measured HRS value with a 20 nm thick BPQD layer is 10^8^–10^9^ Ω. The significant decrease in the resistance of the HRS is due to the introduction of an increased amount of conductive BPQDs into the PMMA dielectrics. The ability to tune the SET voltage and the resistance of the HRS by varying the thickness of BPQD layer is attributed to the easier formation of the metallic filament in the thicker layer, hence, an increase in the amount of effective charge trapping sites in the thicker BPQD layer. During the SET process, more electrons are attracted and stored, leading to a much easier formation of the metallic phase and a drop in SET voltage in a higher internal electrical field. However, dissipation of the trapped electrons originating from the lateral leakage may result in shorter retention time in the thicker BPQD layer‐based RRAM, which entails a tradeoff between the SET voltage and the retention time.

**Figure 5:**
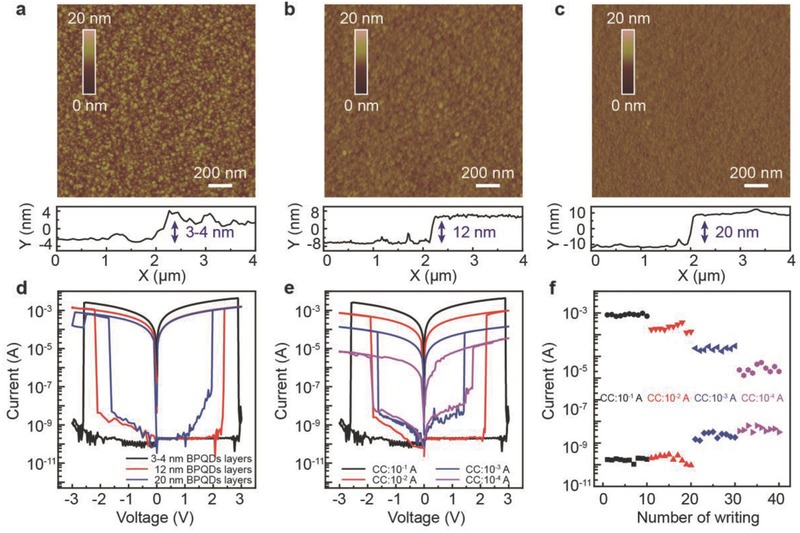
Multilevel data storage of flexible BPQD‐based memory device. Tapping mode AFM images of a) 3–4 nm BPQD layer, b) 12 nm BPQD layer, and c) 20 nm BPQD layer with height profile. d) Current response of BPQD‐based RRAMs to various thicknesses of the BPQD layer. e) *I–V* characteristics of PET/Al/35 nm PMMA/BPQDs/35 nm PMMA/Al cells under four different compliance currents. f) Reversible resistive switching over 40 cycles with different compliance current. The switching pulse duration is fixed to 0.1 s and the reading voltage is 0.5 V.

The *I–V* characteristics of the PET/Al/35 nm PMMA/3–4 nm BPQDs/35 nm PMMA/Al device under several compliance currents are plotted in Figure 5e. By adjusting the compliance current from 0.1 to 10^−4^ A, multilevel reversible resistive switching with non‐negligible fluctuation of SET and RESET voltages were developed. Distinctly diverse resistance states were observed at different magnitude of the compliance currents. The range of the available resistance values is almost four orders of magnitude. As expected, the device displays a bipolar multilevel resistive switching response in both positive and negative voltage cycles. Such response is indicative of bipolar memristor‐like behavior. Compared to the previously reported BP‐based RRAM, our device based on the self‐assembled BPQD thin layer demonstrates superior memory performance with precisely modulated multilevel states and with high fidelity. The distribution of multilevel states upon the number of SET/RESET cycles is displayed in Figure 5f. Distinctive levels of current are well maintained during ten repetitions of voltage pulses. These results reveal that at least four unique states can be realized in the PET/Al/35 nm PMMA/3–4 nm BPQDs/35 nm PMMA/Al device for multilevel information storage purposes. A higher compliance current induces higher conductivity of the LRS, indicating that the tiny filaments regroup in the active layer.^43^ We note a higher compliance current for switching the device to the LRS leads to the formation of a more robust filament, which requires more energy for sequential rupture.^43^, ^44^

In conclusion, we first demonstrated tunable memory characteristics and multilevel data storage in BPQD‐based RRAM in this communication. Solution‐process BPQDs sandwiched between two layers of PMMA dielectrics exhibited electroforming‐free resistive switching behavior with a record ON/OFF ratio of 3.0 × 10^7^ for 2D material‐based RRAMs. By optimizing the thickness of BPQD layer, we were able to tune the SET voltage and the ON/OFF ratio in a predictable manner. We further demonstrated the in situ control of the SET voltage and ON/OFF ratio in the BPQD‐based RRAM with compliance setting, and achieved four‐level data storage by modulating the compliance value from 0.1 to 10^−4^ A. In addition, we demonstrated that the solution‐processed BPQD‐based cell could be reliably built on flexible substrates and perform without degradation under many bending cycles. The results of this study indicate that our BPQD‐based RRAM is very promising with practical applications in the flexible electronics field.

## Experimental Section

*Materials*: PMMA powder (*M*_W_ = 1 20 000) was obtained from Aldrich. Black phosphorus powder was obtained from XFNANO (99.998%).

*BPQDs Synthesis*: Liquid exfoliation assisted by sonication was adopted to synthesize the black phosphorus quantum dots. Specifically, 6 mg black phosphorus powder was transferred into a 40 mL scintillation vial in a nitrogen glovebox. As a strong polar organic solvent, 20 mL NMP (aladdin, 99.9%) was added, yielding a final concentration of 0.3 mg mL^−1^. Vials were tightly capped and wrapped with parafilm to prevent air exposure before placing into a bath sonicator. Sonication exfoliation lasted for 6 h, resulting in a brown suspension of BP‐NMP. Finally, a centrifuge with rotational speed up to 12 000 rpm was employed to produce uniform BPQDs.

*Device Fabrication*: The bottom electrode was prepared by thermal evaporation of 100 nm thick Al on the PET substrate through a shadow mask. Then, a layer of PMMA was spin coated on the substrate and annealed in the nitrogen glovebox at 120 °C. BPQD suspension was drop casted onto the PMMA film, let it to sit for 2 min self‐assembly process before blowing away the excessive suspension with a rubber suction bulb in one direction, then annealed it at 50 °C in an oven overnight. Next, another layer of PMMA was spin coated on the BPQD layer and annealed at 120 °C to form the sandwich structured active layer. Finally, the top electrode was fabricated by thermal evaporating 100 nm thick Al onto the active layer through a shadow mask.

*Characterization*: The BPQDs were characterized by high‐resolution transmission electron microscope (FEI Tecnai F30). Raman spectra of the BPQDs were obtained using a 514 nm micro‐Raman spectrometer (HORIBA LabRAM HR Evolution). The topography of three BPQD films of various thicknesses was measured using atomic force microscope (VEECO Multimode V). The electrical characteristics of all the devices were measured using Agilent 4155C semiconductor analyzer at room temperature in ambient conditions.

## Supporting information

Supplementary
