# Supplementary material for: Black Phosphorus Quantum Dots with Tunable Memory Properties and Multilevel Resistive Switching Characteristics
Source: Adv Sci (Weinh). 2017 Mar 16;4(8):1600435. doi: 10.1002/advs.201600435 (PMC5566243; doi:10.1002/advs.201600435)
Supplement: Supplementary file 1 — Supplementary [file ADVS-4-na-s001.pdf]

Copyright WILEY-VCH Verlag GmbH & Co. KGaA, 69469 Weinheim, Germany, 2017.

ADVANCED SCIENCE
Open Access

Supporting Information

for *Adv. Sci.,* DOI: 10.1002/advs.201600435

Black Phosphorus Quantum Dots with Tunable Memory Properties and Multilevel Resistive Switching Characteristics

Su-Ting Han, Liang Hu, Xiandi Wang, Ye Zhou, Yu-Jia Zeng, Shuangchen Ruan, Caofeng Pan,* and Zhengchun Peng**

Supporting Information

DOI: 10.1002/advs.201600435
Article type: Communication

Black phosphorus quantum dots with tunable memory properties and multilevel resistive switching characteristics

Su-Ting Han,[†] Liang Hu,[†] Xiandi Wang, Ye Zhou, Yu-Jia Zeng, Shuangchen Ruan, Caofeng Pan* and Zhengchun Peng**

[*] Dr. S. T. Han, Prof. C. Pan, Prof. Z. Peng,
College of Optoelectronic Engineering, Shenzhen University, Shenzhen, 518060, P. R. China
E-mail: zcpeng@szu.edu.cn
[*]Dr. L. Hu, Prof. Y. J. Zeng, Prof. S. Ruan
Shenzhen Key Laboratory of Laser Engineering, College of Optoelectronic Engineering, Shenzhen University, Shenzhen, 518060, P. R. China
E-mail: yjzeng@szu.edu.cn
Prof. Y. Zhou
Institute for Advanced Studies, Shenzhen University, Shenzhen, 518060, P. R. China
[*] Dr. X. Wang, Prof. C. Pan
Beijing Institute of Nanoenergy and Nanosystems, Chinese Academy of Sciences, Beijing, 100083, P. R. China
E-mail: cfpan@binn.cas.cn
[†] S. T. Han and L. Hu contributed equally to this work.

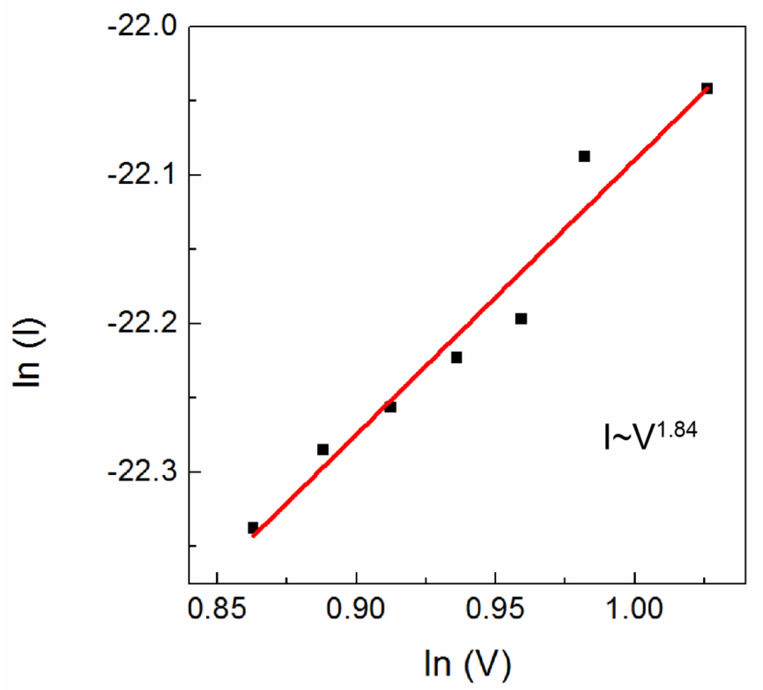

Figure S1. Region I SCLC part of Figure 3a.

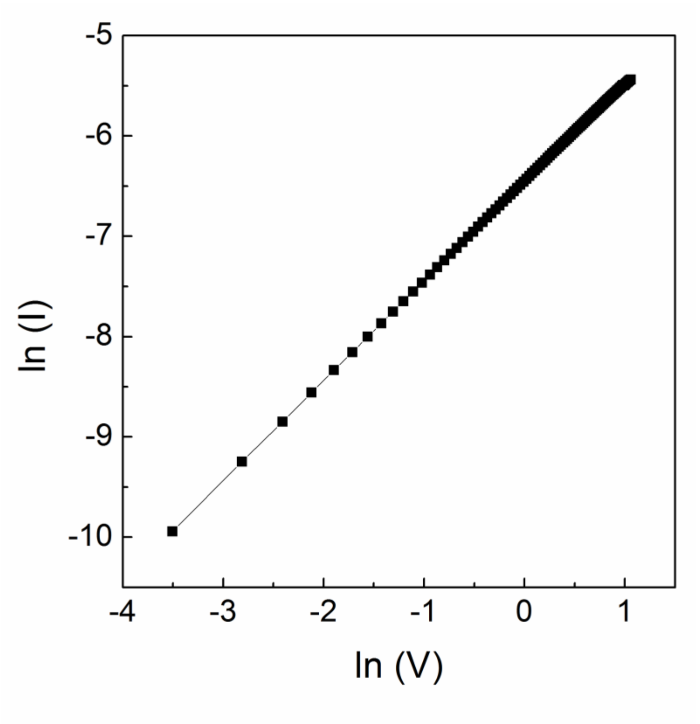

Figure S2. Region III part of Figure 3a.

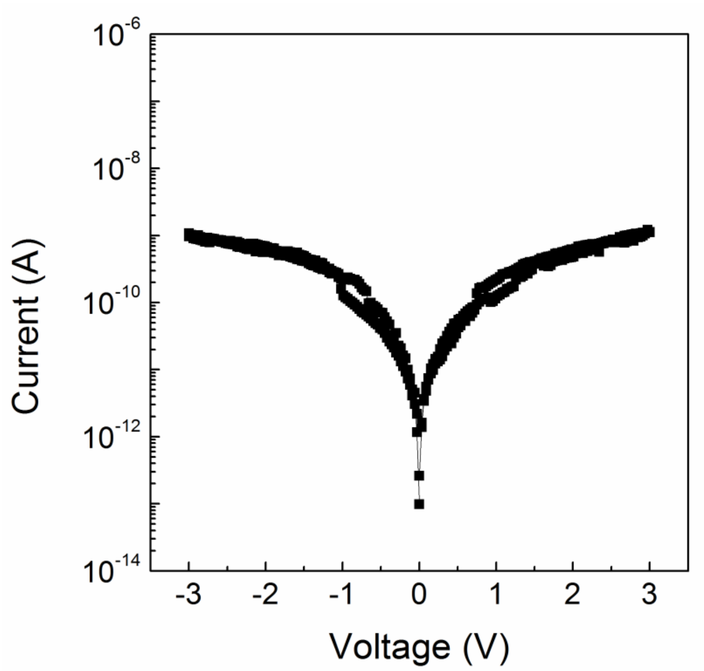

Figure S3. *I*-*V* characteristics of pristine PMMA-based RRAM device.
